# Profile: James Davies

**DOI:** 10.1192/pb.bp.113.046516

**Published:** 2014-08

**Authors:** Neil Armstrong

**Your recent book, *Cracked*, has provoked a lot of interest and comment. For those who haven’t read it, how would you summarise the argument?**

In a nutshell, I argue that psychiatry over the past 40 years, under the dominance of the medical or ‘technological’ model, has done a lot of harm in the name of helping vulnerable people. Not intentionally, I hasten to add, but as an outcome of taking the medical model too far. Your readers will be familiar with the arguments: psychiatry has medicalised more and more natural, albeit painful, responses to the difficulties of living; it has become wedded to medications of questionable value (for many people) and whose long-term effects are still uncertain; it has allowed itself to be compromised by pharmaceutical ties; it has stigmatised people through labels and has sold itself as closer to the rest of medicine than it is. All this has led to a situation in which the integrity and efficacy of the profession is now under serious scrutiny.

**What led you to write the book?**

My experience of working with people in the NHS who had been adversely affected by psychiatric diagnoses and drugs that were, in my view, often entirely unnecessary. It takes relatively little time to assign a descriptive label, but it takes many months to really understand a person and why they suffer. Yet most psychiatrists have little time for the latter, which is why I’d so often encounter understandable human experience, even necessary experience, being medicalised and medicated to the detriment of the patient. In many cases the diagnoses were leading to little other than the illusion of understanding, for doctors, and stigma and self-stigma for patients. The medications themselves, although helpful for some of the more severely distressed in the short term, ended up confusing us all: what experience was drug induced and what the product of the ‘person’ or the ‘condition’? After some time nobody would really know any more, patient, psychologist or doctor.

**The issues that *Cracked* addresses are very much in the public eye - it is as if doubts or concerns about mental healthcare are part of the zeitgeist. Do you have a sense as to why these debates seem so important now?**

These debates are so important because more people than ever before are being affected - directly or indirectly - by psychiatric drugs and diagnoses. So the public is waking up and asking, now wait a minute, do one in four people really have a mental health disorder in any given year? Were there really over 50 million prescriptions of antidepressants in England in 2012? Is it really the case that diagnostic manuals are expanding for no justifiable scientific or clinical reasons? Is there really no convincing evidence of biological markers for most mental disorders? Is it really true that clinical outcomes still disappoint year on year? And then they hear stories about compromising pharmaceutical ties, poor provision for non-medical alternatives, psychiatry’s enduring struggle for ‘medical’ status. Putting two and two together, people are asking the inevitable: to what extent is psychiatry’s attachment to the medical model continuing for non-clinical reasons? To what extent is it merely serving its own, or business, purposes?

**So mental health professionals underestimate the harm they do?**

There is no question in my mind that we underestimate such harm. This is the particular blind spot of all helping professions. Our raison d’être makes it harder for us to spot or admit such harm. Investment bankers probably struggle less with this, because helping people is not primarily their aim, it’s making money. So while a banker may harm someone but still believe himself to be a ‘good banker’, that can’t be said of a doctor. A doctor’s professional esteem is located in his or her capacity to heal, which makes evidence to the contrary harder to brook. This can lead to defensiveness in the face of such evidence.

**Is the size of these harmful effects really greater than the benefits of effective treatments? Is there high-quality evidence to support that claim?**

I offer this notion as a hypothesis that the arguments in *Cracked* oblige us to take very seriously. But now let me flip that question on its head - is there high-quality evidence to support the opposite, that the benefits of the medical model have outweighed its harmful effects? If we consider not just the treatment of the most severely distressed, but of all those affected by psychiatric drugs, diagnoses and beliefs, then I think psychiatry would be hard pushed to produce any such evidence. If you disagree, then show me the evidence.

**Can psychiatry be held responsible for stigma? Does psychiatry not reduce stigma by disseminating knowledge?**

It has tried to do this over the past 20 years or so through anti-stigma campaigns, but these campaigns have less disseminated ‘knowledge’ than ‘information’ which, more often than not, has served the campaigners’ interests. In the name of educating an uncomprehending public (i.e. the supposed cause of stigma), such campaigns have actually ended up doing other things, like making special pleas for more professional services, marketing positive images of psychiatric care and expertise, and promoting self-interested perspectives that are not only highly contestable (for example, that mental illness is like physical illness), but that may have actually driven up rates of stigma. This last point is crucial because the problem with saying mental illness is just like physical illness (a common refrain of campaigns) is that this view is not just unsubstantiated but potentially harmful: research shows that people will treat a person more harshly if they believe their ‘illness’ to be caused by a biological rather than social or psychological problem (a person driven by biology is viewed as less free and predictable, and therefore more dangerous, than ‘ordinary people’). In other words, the application of the biomedical model to the realm of mental health has created in many areas fear in both sufferers and the public. And insofar as these campaigns subtly promote this model, they have to be seen as part of the problem. As anthropologists have long pointed out, this may be why rates of stigma are far higher in high-income countries, where the medical model dominates, than in low- or middle-income countries, where it does not.

**Fig 1:**
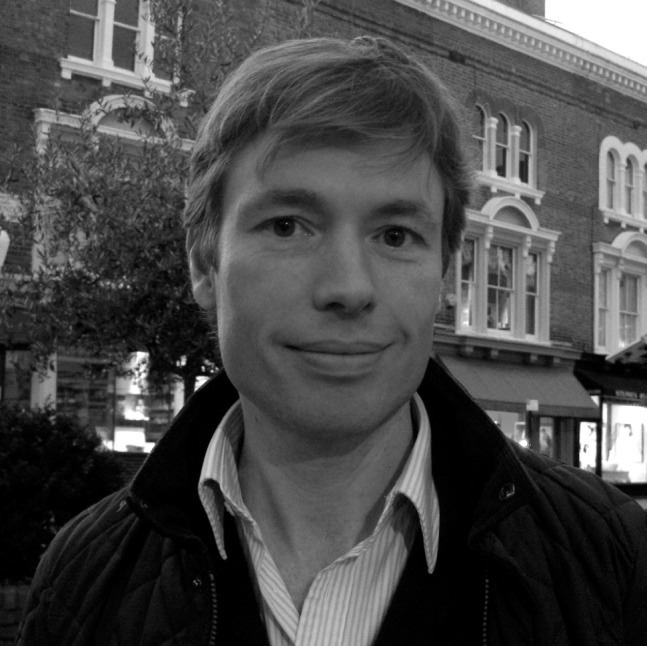
James Davies.

**Your arguments seem to be relevant to people with relatively mild mental health problems. I wonder how you respond to the thought that more seriously unwell people - the people that form the majority of a mental health professional’s case-load - don’t really fit your account.**

The Royal College of Psychiatrists’ president, Sue Bailey, made a similar point when I interviewed her for *Cracked*. She seemed to think that psychiatry was far less responsible for medicalising and medicating people unnecessarily than doctors in primary care. Indeed, GPs do prescribe more drugs, but this is a red herring, because who put these drugs on the map? Who received industry money to promote their value to GPs? And who dramatically expanded the number of mental disorders for which these drugs can be prescribed? But more than this, who championed the medical model while simultaneously undervaluing effective alternatives? Not GPs, of course, but psychiatrists, or at least those psychiatrists who saw in drugs the profession’s salvation. In other words, the argument that *Cracked* has little to do with psychiatry because the book largely focuses on the ‘worried well’ falls down once we acknowledge that biopsychiatry has shaped how we all now understand, manage and respond to our suffering. Psychiatry has altered the zeitgeist -and *Cracked* is about how unjustified and even harmful this alteration has been.

**An objection against some critics of psychiatry is that the criticisms are directed against a straw man - an idealised model of science. According to this view, critics idealise science by supposing that it is something like an impersonal, acultural process that discovers independently existing facts in an unbiased and unmediated fashion. But when examined close up, we know science could never really be like that and psychiatry is no different. This is not really big news. So rather than being a scandal, isn’t the discovery of social processes in scientific method inevitable and unproblematic?**

I agree entirely with your view that no scientific method is entirely a-cultural. What concerns me, however, is the extent to which such bias intrudes in any given research process. Are committees of psychiatrists who vote new disorders into the ICD (and use consensus to decide how such disorders should be defined) reaching conclusions as valid as those attained by way of laboratory experiments? Naturally, they are not. And that’s my point: some kinds of research are simply better at controlling subjective intrusions than others. Psychiatry, with respect to the construction of diagnostic categories, is particularly poor on this front. So poor, in fact, that the DSM and chapter 5 of the ICD should be read predominantly as works of culture. I think more people in psychiatry are beginning to accept this, but such acceptance would shock the public because, for so long, they have been oblivious to just how cavalier and arbitrary the construction of these manuals has been.

**A large part of your criticism is directed towards pharmaceutical companies. How have they influenced psychiatry and how might that be addressed?**

When psychiatry moved out of the asylums it had an opportunity to become truly biopsychosocial, not just in name but also in practice. But that was when the pharmaceutical industry really took hold, leading things to become ever more ‘biobiobio’. I think this has served industry far more than psychiatry, because now that involvement is beginning to backfire. In fact, it is becoming disastrous for psychiatry. It has tarnished the profession more than anything else: psychiatrists have been co-opted into unreported payments, fraudulent research practices and underhand marketing. And, as the Sunshine Act is beginning to make clear, perhaps more than doctors in any other medical specialism. This issue won’t go away. It will dominate in the coming years. And how psychiatry responds is critical. I really hope the College takes the moral lead, and becomes the first UK medical Royal College to demand full transparency (sending requests for members to confess ties is not good enough). The College must create an obligatory online register that documents exactly who gets paid what by whom. Short of that, public perception will continue to dip as more and more comes to light.

**What is the impact of a book like *Cracked?* Does it not just play to the gallery, undermining the profession of psychiatry without helping psychiatrists to do their job? Might it be more constructive to actively engage with debates currently underway within mental healthcare?**

Forgive the word games, but one usual definition of ‘playing to the gallery’ is acting in an exaggerated way in order to appeal to popular taste. But what is the popular taste when it comes to ‘mental illness’? It’s for diagnoses and medications. The arguments in *Cracked* fly in the face of popular taste, and, for that matter, the taste of many working within the mental health sector. I think the best way to help improve our mental health system is not to play to the gallery, but to expose where psychiatry has become misguided by the wrong set of motivations.

**If psychiatry as currently configured is doing more harm than good, what recommendations would you make for change in mental healthcare services? What would the financial implications be if these policies were enacted?**

That’s a large question, but, briefly put, I would argue for more provision for non-medical alternatives in the NHS, greater transparency and accountability with respect to the profession’s financial ties with industry, more critical scrutiny of the disadvantageous effects drugs have for many when taken long term, and finally, for more time spent in training on non-medical alternatives and on learning critical and social/anthropological perspectives. There are many highly thoughtful, critical psychiatrists who have been requesting these things for years, so I am certainly not alone.

